# Shear Thickening Polishing of Quartz Glass

**DOI:** 10.3390/mi12080956

**Published:** 2021-08-13

**Authors:** Qi Shao, Shixiang Duan, Lin Fu, Binghai Lyu, Ping Zhao, Julong Yuan

**Affiliations:** 1 College of Mechanical Engineering, Zhejiang University of Technology, Hangzhou 310014, China; 2112002110@zjut.edu.cn (Q.S.); 2111902075@zjut.edu.cn (S.D.); 2111902024@zjut.edu.cn (L.F.); zhaoping@zjut.edu.cn (P.Z.); jlyuan@zjut.edu.cn (J.Y.); 2 Key Laboratory of Special Purpose Equipment and Advanced Processing Technology, Ministry of Education and Zhejiang Province, Zhejiang University of Technology, Hangzhou 310014, China

**Keywords:** quartz glass, colloidal silica, colloidal cerium oxide, shear thickening polishing

## Abstract

Quartz glass is a typical optical material. In this research, colloidal silica (SiO_2_) and colloidal cerium oxide (CeO_2_) are used as abrasive grains to polish quartz glass in the shear thickening polishing (STP) process. The STP method employs the shear-thickening mechanism of non-Newtonian power-law fluid to achieve high-efficiency and high-quality polishing. The different performance in material removal and surface roughness between SiO_2_ and CeO_2_ slurries was analyzed. The influence of the main factors including polishing speed, abrasive concentration, and pH value on the MRR, workpiece surface roughness, and the surface topography was discussed. Two different slurries can both achieve fine quartz surface in shear thickening polishing with the polishing speed 100 rpm, and pH value 8. The quartz glass surface roughness *R_a_* decreases from 120 ± 10 to 2.3 nm in 14 minutes’ polishing with 8 wt% 80 nm SiO_2_ slurry, and the MRR reaches 121.6 nm/min. The quartz glass surface roughness *R_a_* decreases from 120 ± 10 to 2.1 nm in 12 minutes polishing by 6 wt% 100 nm CeO_2_ slurry and the MRR reaches 126.2 nm/min.

## 1. Introduction

Quartz glass has been widely used in aerospace, high-power lasers, detection system, optical communication, and laser fusion devices due to its advantages of strong resistance to laser damage, low thermal expansion coefficient, good spectral characteristics, and good thermal shock resistance [1]. Modern optical systems have more and more stringent requirements on the surface roughness of optical components. However, quartz glass is a typical material with high hardness and low fracture toughness, which leads to its difficult-to-processing characteristics [2,3]. The traditional lapping and polishing process can achieve the nanometer level of workpiece surface roughness. However, the traditional contact-processing technology mainly uses mechanical action to remove material, which is easy to cause surface/subsurface damage and affect the performance of optical components [4].

In recent years, many polishing methods have been successfully applied to polishing optical parts such as magnetorheological finishing (MRF), ion beam figuring (IBF), chemical mechanical polishing (CMP), and so on. Zhao et al. used IBF to process the quartz wafer, the RMS value of the workpiece surface decreased from 35.598 nm to 5.060 nm after three iterations [5]. CMP greatly improves the polishing efficiency and workpiece surface quality through the chemical and physical effects of the polishing slurry on the optical glass [6]. Wang et al. [7] obtained a good optical glass surface with an RMS 4.7 A° in a 1 mm × 1 mm area by CMP method, and the MRR 675 nm/min was achieved. Yin et al. use MRF to process K9 glass and use a slotted polishing head to obtain a surface with a roughness of 40 nm under optimized processing parameters [8]. Mosavat et al. [9] simulated the deformation of monocrystalline silicon wafers with the magnetic abrasive finishing (MAF) process, and the workpiece surface roughness *R_a_* decreased from 401 nm to 63 nm after processing with optimized parameters. Mosavat et al. [10] studied the effect of process parameters on the reduction rate in the surface roughness of monocrystalline silicon wafers during the MAF process. The research shows that the maximum reduction rate of the silicon wafer is 3.7 nm, and the workpiece surface roughness is 31 nm after processing. Fukushima et al. [11] proposed a new grinding and CMP to remove burrs. Both sides of the silicon wafer were ground and precisely polished after etching to obtain better angular resolution.

The shear-thickening polishing (STP) based on non-Newtonian fluid rheological characteristics was proposed to realize the flexible polishing of the curved surface of the workpiece [12]. A complex cutting edge of cemented carbide insert was polished by STP, and the surface roughness *R_a_* at the cutting edge was reduced from 121.8 nm to 7.1 nm after 15 minutes’ polishing [13]. The surface roughness *R_a_*/*R_z_* of the black LT substrate was reduced rapidly from 200.5/1374.6 to 4.2/22.1 nm after 4 min polishing by the STP method [14]. D. N. Nguyen et al. obtained a good alloy steel SCM435 gears surface with a surface roughness of 13 nm by STP method under optimal machining parameters [15]. M. Li et al. used the adaptive shearing-gradient thickening polishing (AS-GTP) method to improve surface accuracy and restrain subsurface damage on lithium niobite (LiNbO_3_ or LN) crystal. Under certain processing conditions, surface roughness and subsurface damage depth also declined to a minimum critical threshold (<1 nm) [16]. Min Li et al. obtained a super-smooth KDP surface with a surface roughness of 1.37 nm and high shape accuracy by anhydrous-based STP [17]. Binghai Lyu et al. utilized the STP method to achieve high efficiency and high-quality polishing of the concave surface of the high-temperature nickel-based alloy turbine blade. The concave surface roughness *R_a_* of the turbine blade was reduced rapidly from 72.3 nm to 4.2 nm after 9 min polishing [18].

SiO_2_ and CeO_2_ are two kinds of abrasive grains commonly used in the polishing process of quartz glass. The purpose of this article is to clear the different performances of SiO_2_ and CeO_2_ slurries on the material removal mechanism of quartz glass and the chemical reaction between polishing slurry and workpiece, and give a selection reference of slurry for the shear thickening polishing process of quartz glass workpiece. The effects of different concentrations, polishing slurry pH value, and polishing speed on the surface quality and MRR of the workpiece were investigated through experiments.

## 2. Principle of Shear Thickening Polishing

**Figure 1:**
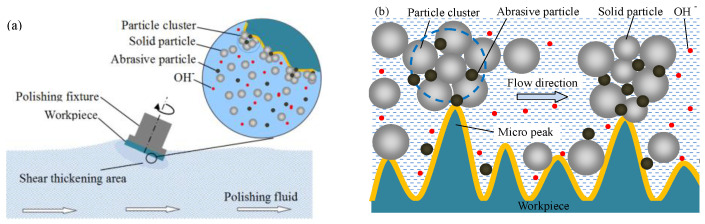
Schematic illustration of STP principle: (**a**) Macroscopic schematic diagram of polishing slurry acting on the workpiece; (**b**) schematic diagram of microscopic material removal.

The macroscopic schematic diagram of the shear thickening polishing of a plane quartz glass workpiece is shown in Figure 1a. The STP slurry is prepared by uniformly dispersing abrasive particles in the base fluid with a shear thickening effect [19]. The rheological properties of the STP slurry change when the shear strain rate applied to the slurry exceeds a critical value. The viscosity of the slurry rises sharply, and the slurry converts to a “flexible fixed abrasive tool” that can adapt to the polishing of various curved surfaces. Although STP can effectively realize the polishing of curved quartz workpieces, such as lenses and hemispherical resonators, the quartz glass plane is selected in this study for the convenience of observation and analysis. The results can provide a reference for the curved workpiece polishing process. The micro schematic illustration of the material removal mechanism of quartz glass in the STP process is shown in Figure 1b. The abrasive particles are wrapped in particle clusters, which are comprised of solid particles as the shear thickening effect is trigged. The solid particle, a kind of organic soft matter, does not affect the removal of workpiece material during the STP process. Under different shear rates, the solid particles have different holding forces on the abrasive particles. As a result, the applied force on the abrasive particle is enhanced dramatically, and the material removal rate is accelerated. At the same time, a soft layer is generated on the workpiece surface by the chemical reaction between quartz glass and the hydroxide ion (OH^−^). The material removal is further improved.

## 3. Experimental Method and Condition

### 3.1. Experimental Process and Conditions

**Figure 2:**
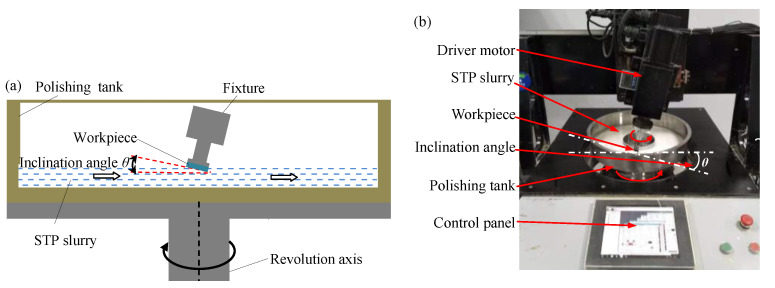
(**a**) Polishing schematic diagram; (**b**) the experimental device of STP.

The research experiments were carried out on the experimental device as shown in Figure 2. The quartz glass was fixed on the fixture. During the polishing process, the workpiece was immersed in the polishing slurry and rotates along the Z-axis to ensure that the workpiece surface can be uniformly polished. It is necessary to ensure that the polishing slurry forms an effective polishing pressure and speed on the workpiece surface, and reduce the speed loss during the polishing process. More importantly, a speed gradient should be generated to apply a shear action on the polishing slurry effectively and produce a thickening effect. Therefore, the inclination angle *θ* between the plane and the horizontal direction is set as 13° [14]. To study the influence of polishing parameters on the surface of quartz glass during the STP polishing process, optimize the polishing parameters and improve the polishing efficiency of quartz glass, the processing conditions are shown in Table 1. The diameter of the quartz glass is 20 mm. The polishing speed and abrasive concentration have been limited in a small variation range according to basic research. Quartz glass undergoes chemical reactions under alkaline conditions, so the polishing effect under the pH values 7, 8, 10, 12 of the polishing slurry was studied. Citric acid and potassium hydroxide were used as pH adjusters. The properties of quartz glass in this study are shown in Table 2. The diameter of the polishing tank is 400 mm, and the polishing speed in this study is defined as the rotation speed of the polishing tank. 

**Figure 3:**
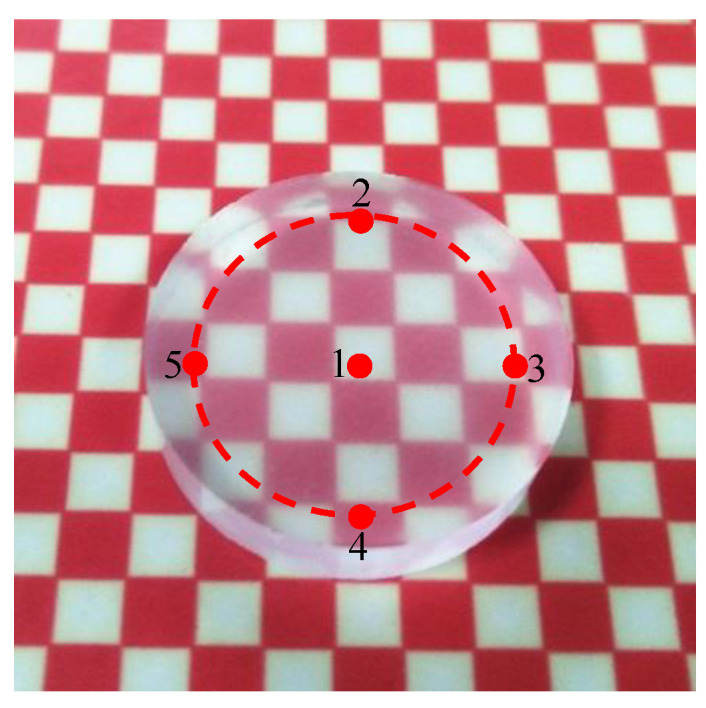
Schematic diagram of observation points on the workpiece surface.

The workpiece surface was observed every five minutes during the polishing process. The roughness was measured at five different positions on the processing surface, as shown in Figure 3, four points on a circle with a diameter of 15 mm and one point at the center of the workpiece surface. The workpiece surface topography was measured by a scanning electron microscope (SU8010, HITACHI) and a large-field-depth digital microscope (VHX-7000). The roughness of the processing surfaces was measured by a Taylor roughness tester (Form Talysurf i-Series 1) and a white light interferometer (Super View W1). Taylor’s sampling length for each measurement point is 2 mm. The sampling range of the white light interferometer is 0.5 × 0.5 mm. An energy dispersive spectrometer (EDS) is used to test the elements on the processed surface. The quality change of the workpiece material before and after polishing was measured by a precision balance (MSA225S-CE) with an accuracy of 0.01 mg. The material removal rate can be calculated by Equation (1).

*H* = Δ*m*/*ρS* (1)

where Δ*m* is the weight loss after polishing, *ρ* is density, *S* is the processing area.

### 3.2. Preparation of STP Slurry

**Figure 4:**
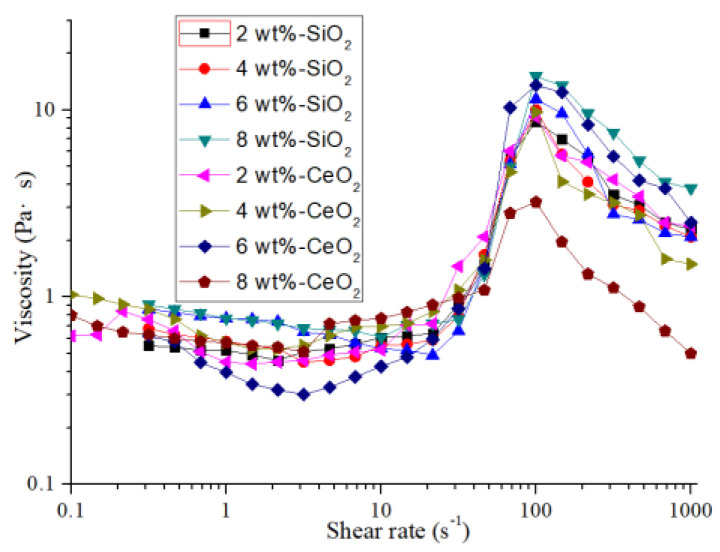
Rheological curves of different kinds of the polishing slurry.

The STP slurry is the key to the STP method. In this research, STP slurry is obtained by uniformly dispersing abrasive particles in a non-Newtonian fluid base fluid, which includes thickening phase polymer and dispersant. It is necessary to stir the slurry for 30 min and disperse it for 15 min by an ultrasonic device to make the slurry uniform. Figure 4 shows the viscosity curve of the STP slurry with different abrasive particle concentrations under different shear rates. All rheological curves were measured by the stress-controlled rheometer (MCR 302, Anton Paar, Graz, Austria), a cone-and-plate (Ø25 mm diameter, 2° cone angle, and 0.103 mm gap) was used, and the testing temperature was controlled at 25 °C by the Peltier heating jacket. Every measurement was repeated three times to quantify the measurement error. There are three viscosity zones at different shear rates which is the same as the viscosity curve of the typical three-stage shear thickening fluid [12]. A slight shear-thinning behavior can be found as the shear rate is low. A strong shear thickening behavior can be found as the shear rate is exceeded and shear-thinning behavior is observed as the shear rate further increases.

## 4. Results and Discussion

### 4.1. Material Removal Mechanism of Quartz Glass with Different Slurry

**Figure 5:**
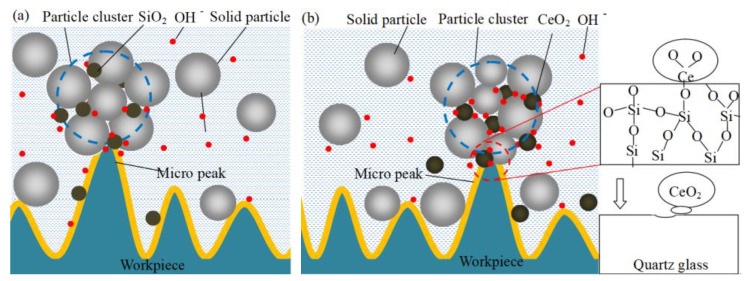
Schematic diagram of the material removal process of quartz glass: (**a**) SiO_2_; (**b**) CeO_2_.

**Figure 6:**
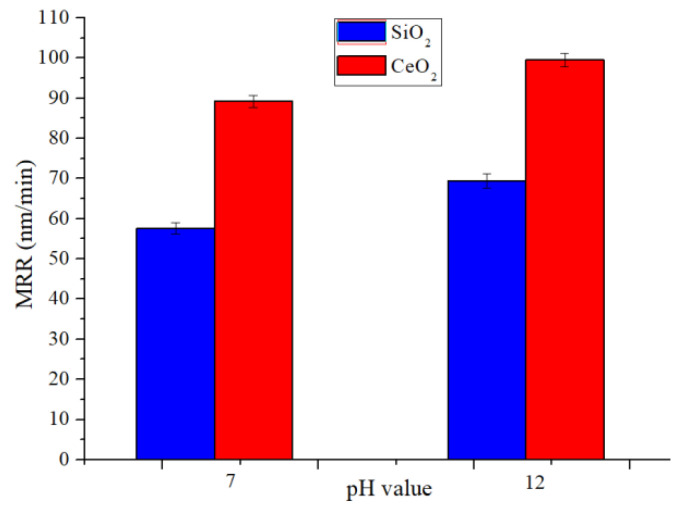
Comparison of the MRR of SiO_2_ and CeO_2_ under conditions with polishing speed 90 rpm and abrasive concentration 6 wt%.

The schematic diagram of the material removal process is shown in Figure 5. The main component of quartz glass is SiO_2_. The Mohs hardness of SiO_2_ is similar to CeO_2_. Quartz glass reacts with water to form silanol in a water environment, and the reaction is shown in Equation (2) [20]. Then the surface reactants and workpiece materials are removed by the mechanical action of SiO_2_ abrasive, as shown in Figure 5a. Polishing under alkaline conditions can improve the MRR because quartz glass can react with OH^-^, and the reaction is shown in Equation (3) [21]. When the polishing speed is 90 rpm, the abrasive concentration is 6 wt%, the MRR of SiO_2_ increased from 57.6 nm/min at pH 7 to 69.4 nm/min at pH 12, the MRR of CeO_2_ increased from 89.2 nm/min at pH 7 to 99.5 nm/min at pH 12, the MRR comparison is shown in Figure 6. Figure 5b presents the process with CeO_2_, it not only shows the removal method of SiO_2_ but also other chemical reactions when CeO_2_ is used for polishing. Cerium hydroxides, the product of cerium atoms and water as shown in Equation (3) [22], will react with silanol to form Ce-O-Si bonds as shown in Equation (4) [22]. The bond energy of Ce-O-Si is greater than the bond energy of Si-O-Si in the quartz glass. With the relative movement of the abrasive particles and the workpiece, the SiO_2_ can be brought out from the quartz glass [23]. During STP processing, the CeO_2_ abrasive surface can adsorb more OH^-^ than the SiO_2_ abrasive because CeO_2_ is more OH^-^ friendly than SiO_2_ [20], as shown in Figure 5. It is more beneficial to promote the chemical reaction between the quartz glass surface and the alkaline to a certain extent when the abrasive grains are in contact with the workpiece surface. Finally, the reactant is taken away from the surface of the material by abrasive particles. In addition, it is also conducive to the stable existence of CeO_2_ particles in the alkaline polishing slurry [23]. Therefore, the MRR of CeO_2_ is higher than SiO_2_ under the same polishing parameters. The MRR comparison is shown in Figure 6, when the experimental conditions are the polishing speed is 90 rpm, the abrasive concentration is 6 wt%, the MRR of CeO_2_ is higher than SiO_2_.

(2) $${\mathrm{SiO}}_{2}+2H_{2}O\to {\mathrm{Si}(\mathrm{OH})}_{4}^{0}$$

SiO_2_ + 2OH^−^ → (SiO_3_)^2−^ + H_2_O (3)

(4) $$\equiv \mathrm{Ce}-O-\mathrm{Ce}\equiv +H_{2}O\to \equiv \mathrm{Ce}-\mathrm{OH}+\mathrm{HO}-\mathrm{Ce}\equiv$$

(5) $$\equiv \mathrm{Ce}-\mathrm{OH}+\mathrm{HO}-\mathrm{Si}\equiv \to \equiv \mathrm{Ce}-O-\mathrm{Si}\equiv {+H}_{2}O$$

### 4.2. Polishing at Different pH Values

The process under different polishing slurry pH values is carried out with the polishing speed 90 rpm and the abrasive concentration 6 wt%.

**Figure 7:**
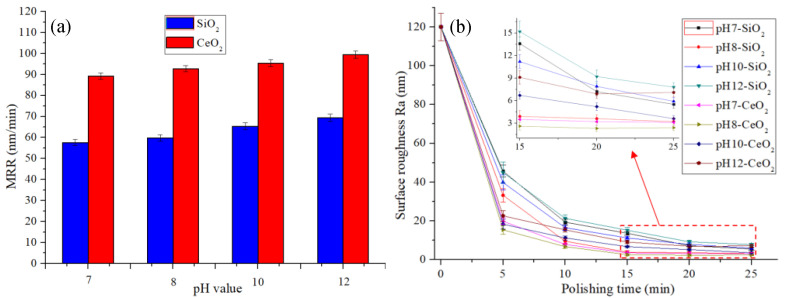
Experimental results conditions with polishing speed 90 rpm and abrasive concentration 6 wt%: (**a**) the MRR of quartz glass, (**b**) the evolution of surface roughness.

**Figure 8:**
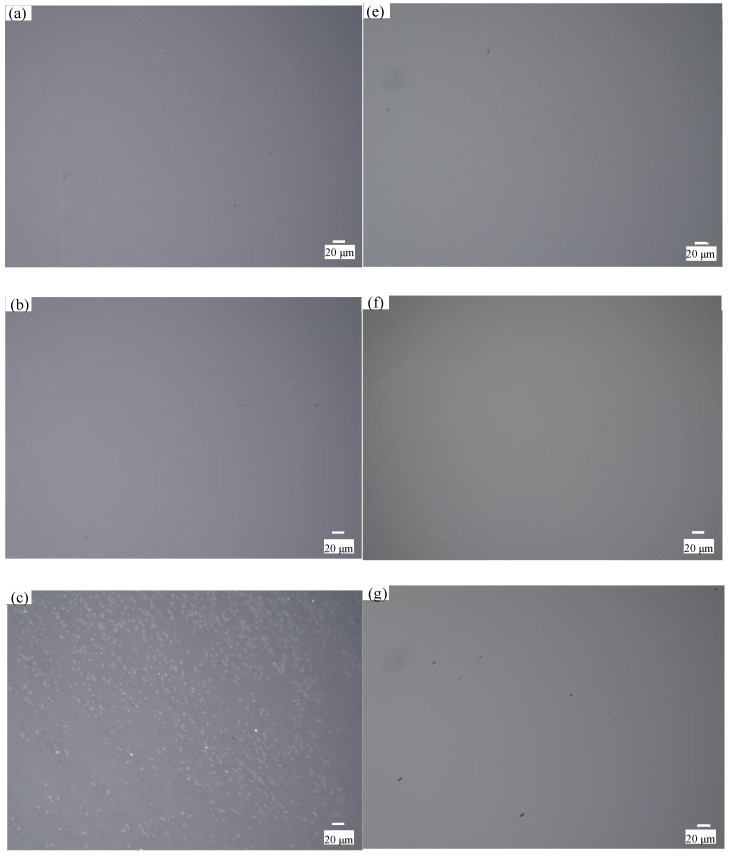

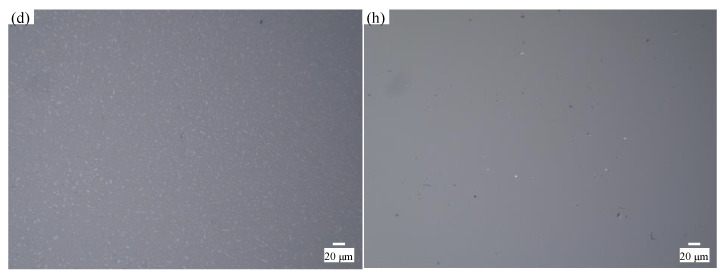
Workpiece surface topography under different slurry pH values: processed by SiO_2_ when (**a**) pH 7; (**b**) pH 8; (**c**) pH 10; (**d**) pH 12; processed by CeO_2_ when (**e**) pH 7; (**f**) pH 8; (**g**) pH 10; (**h**) pH 12.

The MRR of the workpiece during the STP processing is shown in Figure 7a, and the evolution of surface roughness is shown in Figure 7b. It can be seen that the MRR increases as the polishing slurry pH value increases. Under alkaline conditions, the polishing slurry contains a higher concentration of OH^−^, which is beneficial to react with the quartz glass material. Under the same slurry pH value, the MRR of CeO_2_ abrasive particles is higher than SiO_2_. CeO_2_ is more OH^-^ friendly than SiO_2_, which is beneficial to promote the contact of OH^-^ with the workpiece surface and improve the MRR during the polishing. Therefore, CeO_2_ has a higher MRR than SiO_2_ in an alkaline environment. As the polishing slurry pH value increases, the surface roughness of the quartz glass decreases first and then increases. Better surface roughness can be achieved when the pH is 8. When the polishing slurry pH value is too high, the polishing slurry will over corrode the workpiece surface during STP processing which leads to uneven material removal, and pits will appear on the surface after polishing and the surface roughness increases, as shown in Figure 8.

### 4.3. Polishing at Different Speeds

**Figure 9:**
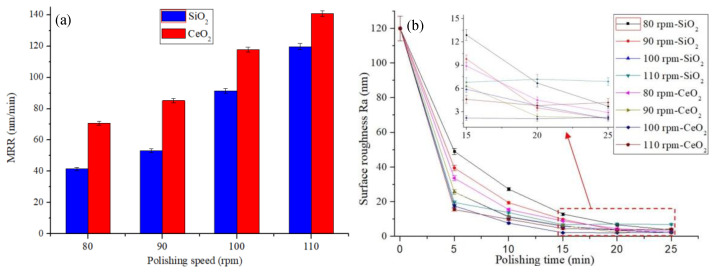
Experimental results under conditions with abrasive concentration 6 wt% and slurry pH value 7: (**a**) the MRR of quartz glass; (**b**) the evolution of surface roughness.

The polishing slurries were prepared with concentrations of 6 wt% SiO_2_ and CeO_2_. The polishing process is performed under the polishing slurry pH value 7. The polishing experiment was carried out at different polishing speeds. Figure 9 shows that the MRR and roughness change at different polishing speeds.

**Figure 10:**
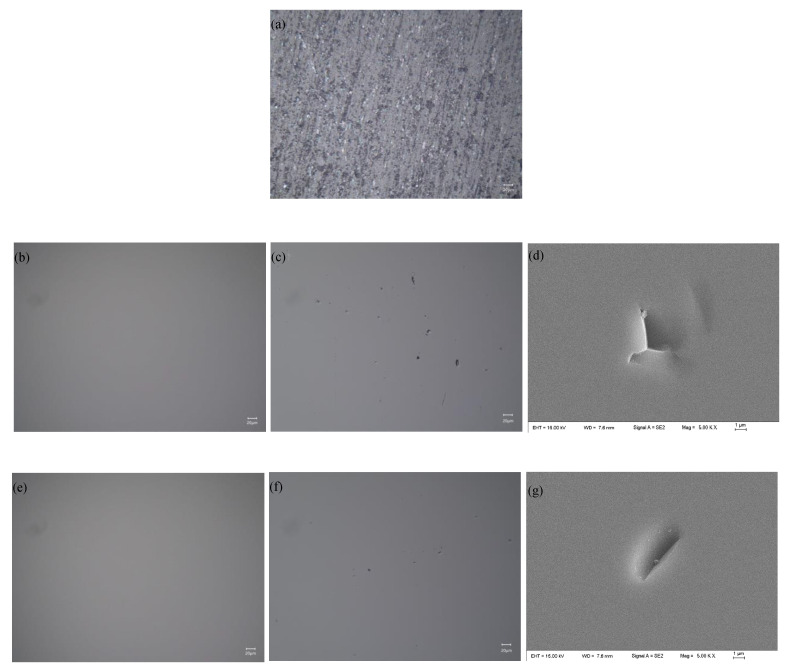
The surface topography under different polishing speed: (**a**) original surface; processed by CeO_2_ when polishing speed is (**b**) 100 rpm; (**c**) 110 rpm; (**d**) SEM topography of pit, processed by SiO_2_ when polishing speed is (**e**) 100 rpm; (**f**) 110 rpm; (**g**) SEM topography of pit.

**Figure 11:**
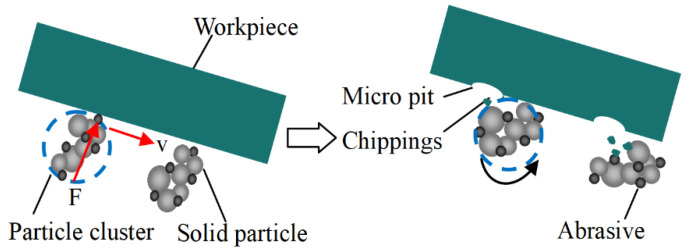
Schematic diagram of pit formation.

Figure 9a shows that the MRR greatly increases as the polishing speed increases, which is due to the shear stress of the polishing slurry on the workpiece increases as the polishing speed increases. At the same polishing speed, the MRR of CeO_2_ abrasive is higher than that of SiO_2_. This is because the CeO_2_ polishing slurry has a higher viscosity and has a higher ability to hold abrasive grains than SiO_2_ polishing slurry at the same shear rate. Figure 9b shows that the surface roughness decreases as the speed increases, and the surface roughness increases when the polishing speed is 110 rpm. Figure 10 shows the workpiece surface topography after 20 minutes’ polishing by SiO_2_ and 15 minutes’ polishing by CeO_2_ when the polishing speed is 100 rpm and 110 rpm. There is almost no defect on the polished surface when the polishing speed is 100 rpm, as shown in Figure 10b,e. When the polishing speed is 110 rpm, there will always be some pits on the processed surface, as shown in Figure 10c,f. The SEM topography of pits is shown in Figure 10d,g. The schematic diagram of pit formation is shown in Figure 11. The pressure *F* and polishing speed *v* applied on the workpiece surface by the particle clusters, and there are translational and rotational movements during the polishing process. When the polishing speed increased to 110 rpm, the *F* applied by the particle clusters on the workpiece surface exceeds the brittle fracture value of quartz glass, and the particle clusters are pressed into the workpiece surface like an indenter, causing brittle damage and forming pits on the workpiece surface.

### 4.4. Polishing at Different Concentrations

The polishing slurries were prepared with concentrations of 2 wt%, 4 wt%, 6 wt%, and 8 wt% SiO_2_ and CeO_2_. The polishing process is performed under the polishing slurry pH value of 7, and polishing speed of 90 rpm.

**Figure 12:**
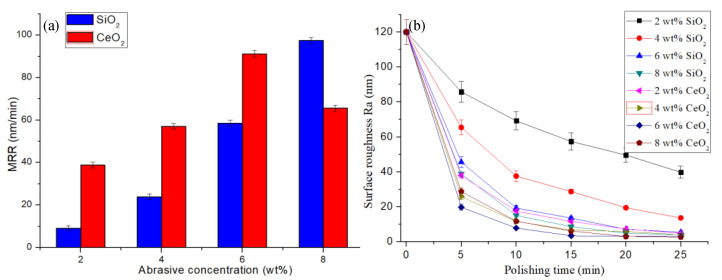
Experimental results under conditions with slurry pH value 7, and polishing speed 90 rpm: (**a**) the MRR of quartz glass; (**b**) the evolution of surface roughness.

The MRR of the workpiece during the STP processing is shown in Figure 12a, and the evolution of surface roughness is shown in Figure 12b. As the concentration of abrasive particles increases, the number of abrasive particles acting on the workpiece surface increases, and the MRR increases. The MRR of the CeO_2_ abrasive particles is higher than the SiO_2_ abrasive particles when the abrasive concentration is 2 wt% to 6 wt%. As shown in reaction Equations (2)–(4), there is a certain amount of adsorption removal when using CeO_2_ abrasive grains to process quartz glass. The material removal is mainly achieved by mechanical action during quartz glass processing by SiO_2_ abrasive. Therefore, under the same abrasive grain concentration, the polishing efficiency of CeO_2_ abrasive grains is higher than SiO_2_ abrasive grains, and the workpiece surface roughness is lower. When the concentration of abrasive particles is 8%, the fluidity of the polishing slurry prepared by CeO_2_ is weakened, and the thickening strength is declined. The high concentration of CeO_2_ causes hydrolysis of polyhydroxy aldehyde polymers leading to changes in rheological properties. The viscosity curve of the STP slurry is shown in Figure 4. During the polishing process, the shear thickening effect of the polishing slurry decreases sharply which leads to low holding force on the CeO_2_ particles and the MRR decreases. The polishing effect is lower than that of SiO_2_.

### 4.5. Polishing Experiment with Selected Parameters

**Figure 13:**
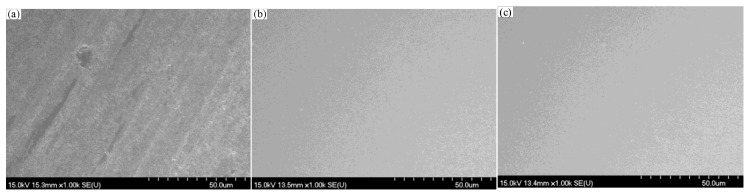
SEM topography of workpiece surface (**a**) before polishing; (**b**) polishing by SiO_2_; (**c**) polishing by CeO_2_.

**Figure 14:**
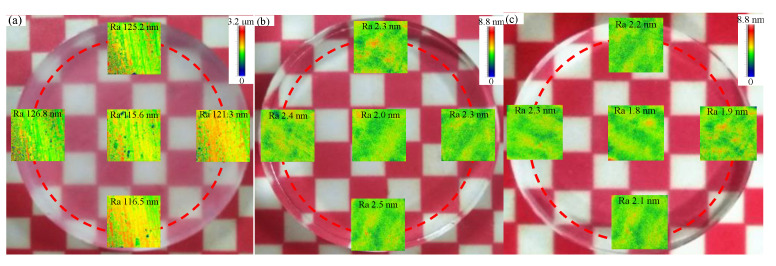
The surface contrast of the quartz glass (**a**) before polishing; (**b**) after polishing by SiO_2_; (**c**) after polishing by CeO_2_.

It can be drawn from Section 4.2 and Section 4.3 that better surface roughness can be obtained with the polishing slurry pH 8, and a higher material removal rate and surface quality can be obtained with the polishing speed 100 rpm. It also indicates from Section 4.4 that a better polishing effect can be achieved with the 8 wt% concentration of SiO_2_ slurry or the 6 wt% concentration of CeO_2_ slurry. The optical quartz glass was polished under the selected conditions with the polishing speed 100 rpm, and the slurry pH value 8. The workpiece surface roughness Ra decreased from 120 ± 10 nm to 2.3 nm in 14 min and the MRR reaches 121.6 nm/min by using 8 wt% SiO_2_. The workpiece surface roughness Ra decreased from 120 ± 10 nm to 2.1 nm in 12 minutes’ polishing by 6 wt% CeO_2_ and the MRR reaches 126.2 nm/min. The workpiece surface scanning electron microscope (SEM) topography before and after polishing is shown in Figure 13. The images of the quartz glass before and after polishing are shown in Figure 14, and a smooth quartz glass surface is obtained.

## 5. Conclusions

The shear thickening polishing experiments of quartz glass with SiO_2_ slurry and CeO_2_ slurry were carried out in this study, and the performance difference between the two slurries and the mechanism was discussed. Based on experimental and the theoretical analysis presented above, the following important conclusions can be drawn: although both slurries can achieve a smooth surface in STP process of quartz materials, the CeO_2_ slurry has a greater MRR and lower surface roughness than SiO_2_ slurry under the same processing condition. The MRR is improved under alkaline conditions, and a better surface can be obtained with pH 8 slurry. There are pits on the workpiece surface making surface roughness increase when the pH value is higher than 8. The reduction rate of surface roughness increases with increasing polishing speed, and also polishing speed applies over high pressure on the workpiece surface causing surface pits. Polishing speed 100 rpm is considered as the optimal value in this study as the MRR and surface quality are evaluated at the same time. A high MRR and low roughness can be achieved with the 8 wt% SiO_2_ slurry or the 6 wt% CeO_2_. The quartz glass was polished under the selected conditions. The surface roughness *R_a_* decreases from 120 ± 10 to 2.3 nm in 14 minutes’ polishing by SiO_2_ slurry and the MRR reaches 121.6 nm/min. The surface roughness *R_a_* decreases from 120 ± 10 to 2.1 nm in 12 minutes’ polishing by CeO_2_ slurry and the MRR reaches 126.2 nm/min. The results show that the STP is a promising efficient polishing method for quartz glass, and the research on the STP process for complex curved surfaces of quartz glass will be carried out.

## Figures and Tables

**Table 1:** Experimental conditions.

Parameters	Values
Abrasive particles	SiO_2_ (80 nm on average), CeO_2_ (100 nm on average)
The diameter of the polishing tank (mm)	400
Inclination angle (°)	13
Polishing speed (rpm)	80, 90, 100, 110
Concentration (wt%)	2, 4, 6, 8
The slurry pH value	7, 8, 10, 12

**Table 2:** Chemical composition and characteristics of quartz glass.

Parameters	Values
Content of SiO_2_ (%)	99.95
Density (g/cm^3^)	2.2
Melting point (°C)	1150
Mohs hardness	7
Poisson ratio ν	0.17
